# Correlation between abdominal visceral fat and the risk of endometrial cancer in patients with polycystic ovary syndrome

**DOI:** 10.4314/ahs.v24i3.22

**Published:** 2024-09

**Authors:** Jiyan Zhang

**Affiliations:** The first department of gynecology, Cangzhou central hospital, Hebei, China

**Keywords:** Polycystic ovary syndrome, Abdominal visceral fat, Endometrial cancer, Occurrence risk

## Abstract

**Objective:**

To explore the correlation between abdominal fat and the occurrence risk of endometrial cancer (EC) in patients with polycystic ovary syndrome (PCOS).

**Methods:**

The clinical information of 120 PCOS patients receiving treatment in our hospital from March 2019 to April 2022 were included in this study. Patients were divided into two groups, endometrial cancer (EC, n=35) and normal group (NM, n=85). Statistical analysis included t-test, c2-test, and Pearson's correlation coefficient. We analysed the data using logistic regression. The predictive accuracy and discriminative ability of the prediction model were assessed by the area under the receiver operating characteristic (ROC) curve (AUC) and calibration curves.

**Results:**

The incidence rate of EC in women with PCOS is 10.91% (12/110). Significant differences were found in waist circumference, hypertension, diabetes, hyperlipidemia, body mass index (BMI), waist-hip ratio (WHR), insulin resistance index (HOMA-IR), visceral fat area (VFA) oestrogen receptor, progesterone receptor, human epidermal growth factor receptor-2 (HER2), estradiol (E2), and luteinizing hormone (LH) between the two groups (P<0.05). No statistical difference was found in age, hip circumference, menopause, use of intrauterine device, progesterone (P) and follicle-stimulating hormone (FSH) between the groups (P>0.05). Multivariate logistic regression analysis showed that BMI, HOMA-IR, VFA and HER2 were independent influencing factors of EC in PCOS patients (P<0.05). The AUC of BMI, HOMA-IR, VFA, HER2 were 0.878 (95%CI: 0.810~0.946), 0.831 (95%CI: 0.751~0.911), 0.816 (95%CI: 0.704~0.929) and 0.737 (95%CI: 0.634,0.840), respectively. The model had more diagnostic effectiveness (AUC=0.973).

**Conclusions:**

In PCOS disease, high-level BMI, HOMA-IR, VFA, and positive HER2 show an increased risk in the incidence of EC. These findings suggest that BMI, HOMA-IR, VFA, and HER2 are potential markers for Risk assessment of EC.

HER2: Human epidermal growth factor receptor-2, E2: estradiol, P: progesterone, LH: Luteinizing hormone, FSH: Follicle-stimulating hormone.

## Introduction

Polycystic ovary syndrome (PCOS) is one of the most common endocrine diseases, with an incidence of 5%-15% 1. Typical features include ovulation disorders, menstrual disorders, and polycystic-like changes in the ovaries 2, and these are closely associated with an increased risk of insulin resistance, endometrial hyperplasia, and carcinogenesis 3. Endometrial cancer (EC) is a common malignant tumor, which often occurs in women of child-bearing age 4. The prevalence rate of EC has been increasing in recent years with the increasing obesity. Medium-quality data statistics show the EC mortality rate will increase by a further 19% within 20 years 5. PCOS is thought to be linked with an elevated risk of tumors, of which EC has been the most frequently reported. Risk factors for EC in PCOS include obesity, ovulation disorders, and insulin resistance^6^, ^7^. According to the report, PCOS is 2.7 to 3 times more likely to develop EC than normal women 8, 9. A meta-analysis 10 indicates that women younger than 54 had a threefold increased risk of EC.

PCOS patients are often associated with obesity, which is a risk factor for the increased risk of EC 11. Adipose tissue can synthesize adipokines, biologically active cytokine peptides that interfere with insulin resistance and lipolysis pathways, causing metabolic abnormalities and carcinogenesis in PCOS patients 12. Although it is well known that obesity is linked with the risk of various diseases and cancers, the correlation between obesity and EC risk in PCOS patients is still unclear, and only a few studies have carried out preliminary exploration 13, 14. Abdominal visceral fat area (VFA) is closely associated with EC overall survival 12, However, there are few studies 11 15 on the relationship between VFA and EC risk, and the relevance between EC incidence and VFA in PCOS is unknown. So, we carried out this study to compare and analyse abdominal VFA between EC patients and non-EC patients in PCOS patients, to explore the correlation between abdominal VFA and EC risk in PCOS patients, and to supplement a reliable basis of the prevention of EC in those who diagnose with PCOS.

## Methods

### Patients

A total of 120 women with PCOS who receive treatment in our hospital (March 2019 - April 2022) were included in this study. According to EC diagnostic criteria, all participants were separated into the endometrial cancer (EC, n=35) and the normal group (NM, n=85). There are 63 patients with normal endometrium, 14 patients with simple positive hyperplastic endometrium, and 8 with atypical hyperplastic endometrium in the NM group.

**Inclusion criteria:** (1) All patients were primary PCOS by definite pathological diagnosis; (2) No hormone therapy in the past three months; (3) The treatment cooperation of the patient was good.

**Exclusion criteria:** (1) Patients with cardiopulmonary function, liver, and kidney function impairment; (2) Patients accompanied by hyperthyroidism, hypothyroidism, and other endocrine diseases; (3) Patients with hormone-related tumors or other severe malignant tumors; (4) Patients with incomplete clinical data. The patients and their relatives were aware and agree and signed informed consent. The local medical ethics committee has reviewed and approved the study.

### Baseline data

Baseline data included age, the circumference of the waist and hip, waist-to-hip ratio (WHR), body mass index (BMI), age at menarche, menopause, and the history of hypertension, diabetes, hyperlipidemia, and use of intrauterine device.

### Laboratory tests

Venous blood was collected in the morning under a fasting state to determine fasting blood glucose (FPG) and fasting insulin (FINS). The homeostasis model of HOMA-IR, which is the insulin resistance index, is calculated based on FPG and FINS. Visceral fat area (VFA) was measured with an Omron visceral fat (Omrondual scan HDS-2000) measuring device. Oestrogen receptor, and progesterone receptor status was routinely measured using the Allred scoring system 25. HER2 status was evaluated using immunohistochemistry (IHC). The sexual hormone includes estradiol (E2), progesterone (P), luteinizing hormone (LH), and Follicle-stimulating hormone (FSH).

### Statistical analysis

Statistical analysis was conducted by SPSS version 23 software. The continuous variables were displayed as mean ± standard deviation. Count variables were represented by n (%). The t-test and x^2^ test were performed to evaluate the difference between EC and NM groups in clinical characteristics. The correlation between demographic and clinical characteristics was assessed by Pearson's correlation coefficient. The risk factors of EC in PCOS were analysed by logistic regression. Univariate logistic analysis was used to screen out the variables with differences, and then the variables selected were included in the regression model. Receiver operating characteristic curve (ROC) was used to evaluate the prediction sensitivity and specificity of the model. It was performed with the function of ROC analysis in the SPSS. ROC reflects the correlation between sensitivity and specificity. The prediction accuracy was represented by the area under curve (AUC), which was calculated by the function of ROC analysis in the SPSS/span>. It is generally believed that 0.5 <AUC≤0.7 suggests poor prediction ability, 0.7 <AUC≤0.9 implies better predictive ability, and AUC> 0.9 indicates high predictive value. P <0.05 was regarded as a significant difference.

## Results

The clinical features of the EC (n = 35) and NM groups (n = 85) are outlined in Table 1. The incidence rate of EC in women with PCOS is 10.91% (12/110). Significant differences were found in the proportions of hypertension, diabetes, hyperlipidemia, BMI, WHR, HOMA-IR, VFA, oestrogen receptor, progesterone receptor, HER2, E2, and LH between the two groups (P<0.05). The mean BMI in EC and NM were 26.12 ± 1.67 and 22.88 ± 2.34, respectively. Of the 35 patients of EC, 21 (60.00%) had a history of hypertension, and 23 (65.71%) patients with diabetes. The proportion of patients with hyperlipidemia was much higher than the NM group (62.86% vs 35.29%). No significant difference was found in age, waist circumference, hip circumference, menopause, use of intrauterine device, P and FSH between the groups (P>0.05). The Pearson's correlation coefficient of BMI with waist circumference, hip circumference, waist-hip ratio, and VFA is shown in Table 2. The VFA was significantly positive-related waist circumference, and BMI.

**Table 1:** Clinicopathological characteristics

Variable	EC(n=35)	NM(n=85)	t/*χ*^2^	P
Age (years)	45.71±6.92	47.88±7.69	1.443	0.152
Waist circumference (cm)	81.57±5.21	78.92±4.59	-2.761	0.070
Hip circumference (cm)	103.10±3.42	102.65±0.54	-0.776	0.443
Menopause	15(42.86%)	49(57.65%)	2.179	0.140
Menarche age (years)	13.17±1.21	12.95±0.97	-1.025	0.308
Hypertension	21(60.00%)	32(37.65%)	5.023	0.025
Diabetes	23(65.71%)	38(44.71%)	4.378	0.036
Hyperlipidemia	22(62.86%)	30(35.29%)	7.670	0.006
BMI (kg/m^2^)	26.12±1.67	22.88±2.34	-8.507	<0.001
WHR	0.79±0.05	0.77±0.05	-2.449	0.016
HOMA-IR (mmol/L)	2.36±0.17	2.11±0.21	-6.303	<0.001
VFA (cm^2^)	98.11±21.18	78.63±6.70	-5.333	<0.001

Use of intrauterine device	19(54.29%)	42(49.41)	0.081	0.776

Oestrogen receptor	25(71.43%)	22(25.88)	19.716	<0.001

Progesterone receptor	26(74.29%)	25(29.41%)	18.634	<0.001

HER2	24(68.57%)	18(21.18%)	22.440	<0.001
E2(pmol/L)	98.86±37.53	128.97±50.96	3.578	0.001

P(nmol/L)	2.65±0.31	2.74±0.30	1.412	0.161

LH(U/L)	36.86±6.05	33.51±3.15	-3.102	0.003

FSH(U/L)	25.03±11.14	26.82±11.92	0.785	0.447

BMI: body mass index, VFA: visceral fat area, HER2: Human epidermal growth factor receptor-2, E2: estradiol, P: progesterone, LH: Luteinizing hormone, FSH: Follicle-stimulating hormone.

**Table 2:** Correlation between BMI, waist circumference, hip circumference, and VFA in patients with PCOS

	Waist circumference	Hip circumference	BMI	WHR	VFA
Waist circumference (cm)	1				
Hip circumference (cm)	0.203*	1			
BMI (kg/m^2^)	0.074	0.010	1		
WHR	0.952**	-0.106	0.075	1	
VFA (cm^2^)	0.170	0.236**	0.345**	0.101	1

* Denotes P<0.05

** denotes P<0.001

The logistic regression model was constructed and the assignment of variables was shown in Table 3. Firstly, regression analysis was carried out for each variable, and the results are shown in Table 4. Waist circumference, hypertension, diabetes, hyperlipidemia, BMI, HOMA-IR, WHR, VFA oestrogen receptor, progesterone receptor, HER2, E2, and LH showed statistical differences (P<0.05). Age, waist circumference, hip circumference, menopause, use of intrauterine device, P and FSH were not significantly correlated with EC in this study population (P>0.05).

**Table 3:** The assignment of variables

Variable	Assignment
Age	Continuous variables
Waist circumference	Continuous variables
Hip circumference	Continuous variables
Menopause	No=0, Yes=1
Menarche age	Continuous variables
Hypertension	No=0, Yes=1
Diabetes	No=0, Yes=1
Hyperlipidemia	No=0, Yes=1
BMI	Continuous variables
WHR	Continuous variables
HOMA-IR	Continuous variables
VFA	Continuous variables

Use of intrauterine device	No=0, Yes=1

Oestrogen receptor	Negative=0, Positive=1

Progesterone receptor	Negative=0, Positive=1
HER2	Negative=0, Positive=1
E2	Continuous variables
P	Continuous variables
LH	Continuous variables

FSH	Continuous variables

**Table 4:** Univariate logistic regression analysis

Variable	β	SE	Wal	P	OR (95%CI)
Age (years)	-0.039	0.028	2.05	0.152	0.961(0.911,1.015)
Waist circumference	0.116	0.044	6.872	0.009	1.123(1.030,1.225)
Hip circumference	0.13	0.111	1.375	0.241	1.139(0.917,1.415)
Menopause	-0.596	0.406	2.155	0.142	0.551(0.249,1.221)
Menarche age	0.203	0.198	1.049	0.306	1.225(0.831,1.806)
Hypertension	0.91	0.411	4.895	0.027	2.484(1.109,5.563)
Diabetes	0.863	0.418	4.272	0.039	2.371(1.046,5.374)
Hyperlipidemia	1.132	0.417	7.372	0.007	3.103(1.370,7.025)
BMI	0.797	0.157	25.784	<0.001	2.218(1.631,3.017)
WHR	0.11	0.047	5.568	0.018	1.117(1.019,1.224)
HOMA-IR	0.669	0.138	23.466	<0.001	1.952(1.489,2.559)
VFA	0.116	0.025	22.337	<0.001	1.123(1.070,1.178)

Use of intrauterine device	0.195	0.403	0.235	0.628	1.216(0.552,2.677)

Oestrogen receptor	1.968	0.449	19.245	<0.001	7.159(2.971,17.250)

Progesterone receptor	1.936	0.454	18.180	<0.001	6.933(2.847,16.885)

HER2	2.094	0.451	21.604	<0.001	8.121(3.358,19.642)

E2	-0.016	0.006	8.079	0.004	0.984(0.973,0.995)

P	-0.948	0.676	1.965	0.161	0.388(0.103,1.459)
LH	0.179	0.051	12.298	<0.001	1.196(1.082,1.322)

FSH	-0.013	0.017	0.588	0.443	0.987(0.954,1.021)

According to the results of univariate regression analysis and clinical experience, waist circumference, hypertension, diabetes, hyperlipidemia, BMI, WHR, HOMA-IR,VFA, use of intrauterine device, oestrogen receptor, progesterone receptor, HER2, E2, and LH were included in the multivariate regression model. As shown in Table 5, the independent risk factors for EC in PCOS contained BMI, HOMA-IR,VFA and HER2 (P<0.05). The goodness of fit test of Hosmer-Lemeshow showed that *X*^2^=2.293, P=0.971.

**Table 5:** Multivariate logistic regression analysis

Variable	β	SE	Wald	P	OR (95%CI)
Waist circumference (cm)	-0.457	0.459	0.991	0.319	0.633(0.257,1.557)
Hypertension	1.172	1.173	0.998	0.318	3.227(0.324,32.156)
Diabetes	-0.181	1.488	0.015	0.903	0.835(0.045,15.438)
Hyperlipidemia	1.653	1.54	1.153	0.283	5.225(0.256,106.828)
BMI (kg/m^2^)	1.438	0.564	6.5	0.011	4.213(1.395,12.729)
WHR	58.244	46.651	1.559	0.212	1.972E+25(0,1.009E+65)
HOMA-IR (mmol/L)	6.814	3.207	4.514	0.034	910.703(1.695,489190.279)
VFA (cm^2^)	0.102	0.038	7.309	0.007	1.108(1.028,1.193)
Use of intrauterine device	-1.712	1.264	1.835	0.176	0.181(0.015,2.149)
Oestrogen receptor	0.591	1.097	0.29	0.59	1.805(0.21,15.514)
Progesterone receptor	-1.831	1.471	1.55	0.213	0.16(0.009,2.864)
HER2	2.976	1.217	5.986	0.014	19.614(1.807,212.841)
E2	-0.023	0.016	2.089	0.148	0.977(0.946,1.008)

LH	0.123	0.134	0.847	0.357	1.131(0.87,1.47)

Intercept	-72.428	23.429	9.557	0.002	-

**Figure 1:**
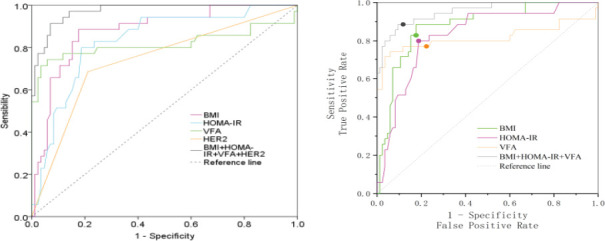
ROC curve of BMI, HOMA-IR and VFA in predicting EC in patients with PCOS

ROC was performed base on a new model constructed by the above independent risk factors (Logistic model=-46.420+0.922BMI+6.209*HOMA-IR+0.087*VFA+2.566*HER2). The AUC of BMI, HOMA-IR, VFA, HER2 were 0.878 (95%CI: 0.810~0.946), 0.831 (95%CI: 0.751~0.911), 0.816 (95%CI: 0.704~0.929) and 0.737 (95%CI: 0.634,0.840), respectively. The AUC of logistic model was 0.973 (95%CI: 0.950~0.997) (Figure 1 and Table 6).

**Table 6:** ROC characteristics of BMI, HOMA-IR, VFA and HER2 in predicting EC in patients with PCOS

Test result variable	Cutoff	AUC	P	Sensitivity	Specificity	95%CI
BMI (kg/m^2^)	24.575	0.878	<0.001	0.886	0.824	(0.810,0.946)
HOMA-IR (mmol/L)	2.285	0.831	<0.001	0.800	0.812	(0.751,0.911)
VFA (cm^2^)	86.35	0.816	<0.001	0.743	0.941	(0.704,0.929)
HER2	-	0.737	<0.001	0.686	0.788	(0.634,0.840)
BMI+HOMA-IR+VFA+HER2	0.336	0.973	<0.001	0.914	0.929	(0.950,0.997)

## Discussion

Previous studies 6, 8, 9 have shown that PCOS prompts the prevalence rate of EC, as obesity, was recognized as one of the risk factors for EC. Current studies on the effect of visceral obesity on EC have focused on the prognosis of EC patients 16, 17, but the relationship between EC risk and EC risk is still unclear. PCOS patients are usually associated with obesity and insulin resistance, and excessive fat leads to chronic estrogen exposure or lack of progesterone, which in turn leads to endometrial hyperplasia and increases EC risk 18. The high expression of insulin-like growth factor (IGF) in endometrial tissues promotes endometrial hyperplasia and leads to the growth and proliferation of endometrial cancer cells 19. Therefore, patients with PCOS are at high risk for EC. Celik et al. 16 reported that VFA may be an important marker for predicting the prognosis of EC, but its diagnostic effectiveness in predicting the incidence of EC in PCOS patients needs to be further explored.

Our study indicated that the risk factors for endometrial cancer in PCOS patients were BMI, HOMA-IR, VFA, and HER2. Obesity is characterized by weight gain and large accumulation of fat, and its related measurement indicators include waist circumference, hip circumference, WHR, BMI, VFA, etc. VFA is a common indicator to measure visceral obesity. Previous studies have demonstrated that high-level VFA leads to an increased risk of various diseases, such as hypertension, diabetes, hyperlipidemia, etc 20, 21, and increase the incidence of EC^22^. The accumulation of fat in PCOS patients promotes the synthesis and secretion of large amounts of estrone, which in turn increases the risk of endometrial cancer 23. Wiwatpanit et al. 24 researched the mechanism related to the risk of PCOS and EC by establishing stent-free multicellular endometrial organoids and found that excessive androgens promoted cell proliferation in endometrial organoids. Ferreira et al. 25 suggested that abnormal endometrial cell proliferation was caused by a lack of estrogen and progesterone withdrawal, and fat accumulation promotes hormonal dysregulation was observed in PCOS. These studies revealed that the association between PCOS and EC is well explained by the association of both diseases with obesity.

In this study, the BMI of the EC group was in the overweight range, and the VFA of the EC group was significantly higher than that of the NM group, suggesting that a high-level VFA is a common characteristic of EC in PCOS patients. Freuer et al. 26 identified the formation and development of EC are affected by fat content, and about 50% of patients with PCOS are overweight or obese, and most of them are characterized by excessive accumulation of abdominal fat 27. Abdominal fat improves the risk of diabetes by inhibiting insulin production by β cells. And adipocytokines produced by abdominal fat itself, such as leptin and adiponectin, disturb the insulin signaling pathway and cause insulin resistance 28. HOMA-IR reflects the degree of insulin in the patient, with higher values suggesting higher levels of insulin resistance. Here, we found that HOMA-IR was significantly higher in the EC than in the NM, suggesting that the higher the degree of insulin resistance, the higher the risk of EC in PCOS patients. There appears to be a higher incidence in patients with obesity and high-level HOMA-IR. The prevalence of metabolic syndrome in women with PCOS is associated with obesity, and being overweight or obese increases the risk of metabolic syndrome 29. HER2 has been shown to strongly promote carcinogenesis. The main mechanism of HER2 activation in cancers is the amplification of the HER2 gene, which results in HER2 protein overexpression.

There are several study limitations in our work. First, we did not analyse the changes in body weight and fat distribution, which may be relevant to the formation and development of EC. Second, this study is a single-center retrospective analysis with small sample size. The conclusion is may be not representative. A multi-center and large-scale assist to the establishment of the model and getting more valuable evidence-based evidence. The mechanism of visceral fat for EC in patients with PCOS should be made clear by more information and research.

In conclusion, for PCOS patients, improving the predictive precision of EC and implementing personalized prevention programs are important measures to prevent the occurrence of the disease. Enhancing the prognosis of patients and reducing the economic burden of patients will be an important question for future investigation. This study implied that BMI, HOMA-IR, VFA, and HER2 were independent risk factors for EC in PCOS patients, and patients with high VFA levels showed a higher risk of EC. This model can be used to screen out patients with a high risk of EC in PCOS as early as possible, and take preventive measures in time. The relationship between PCOS and endometrial cancer is complex, and large-scale studies or pooled analyses should be carried out to reveal the association between them.

## References

[1] Rasquin Leon LI, Anastasopoulou C, Mayrin JV (2022). Polycystic Ovarian Disease. StatPearls.

[2] Meier RK (2018). Polycystic Ovary Syndrome. The Nursing clinics of North America.

[3] Reckelhoff JF, Shawky NM, Romero DG (2022). Polycystic Ovary Syndrome: Insights from Preclinical Research. Kidney360.

[4] Crosbie EJ, Kitson SJ, McAlpine JN (2022). Endometrial cancer. Lancet (London, England).

[5] Njoku K, Barr CE, Crosbie EJ (2022). Current and Emerging Prognostic Biomarkers in Endometrial Cancer. Frontiers in oncology.

[6] Palomba S, Piltonen TT, Giudice LC (2021). Endometrial function in women with polycystic ovary syndrome: a comprehensive review. Human reproduction update.

[7] Macut D, Bjekić-Macut J, Rahelić D (2017). Insulin and the polycystic ovary syndrome. Diabetes research and clinical practice.

[8] Ignatov A, Ortmann O (2020). Endocrine Risk Factors of Endometrial Cancer: Polycystic Ovary Syndrome, Oral Contraceptives, Infertility, Tamoxifen. Cancers.

[9] Murugappan G, Li S, Lathi RB (2019). Risk of cancer in infertile women: analysis of US claims data. Human reproduction (Oxford, England).

[10] Barry JA, Azizia MM, Hardiman PJ (2014). Risk of endometrial, ovarian and breast cancer in women with polycystic ovary syndrome: a systematic review and meta-analysis. Human reproduction update.

[11] Nakamura K, Hongo A, Kodama J (2011). Fat accumulation in adipose tissues as a risk factor for the development of endometrial cancer. Oncology reports.

[12] Chen H, Zhang Y, Li S (2021). The Genetic Association of Polycystic Ovary Syndrome and the Risk of Endometrial Cancer: A Mendelian Randomization Study. Frontiers in endocrinology.

[13] De Pergola G, Silvestris F (2013). Obesity as a major risk factor for cancer. Journal of obesity.

[14] Słabuszewska-Jóźwiak A, Lukaszuk A, Janicka-Kośnik M (2022). Role of Leptin and Adiponectin in Endometrial Cancer. International journal of molecular sciences.

[15] Ye S, Wen H, Jiang Z (2016). The effect of visceral obesity on clinicopathological features in patients with endometrial cancer: a retrospective analysis of 200 Chinese patients. BMC cancer.

[16] Celik E, Kizildag Yirgin I, Goksever Celik H (2021). Does visceral adiposity have an effect on the survival outcomes of the patients with endometrial cancer?. The journal of obstetrics and gynaecology research.

[17] Tangen IL, Fasmer KE, Konings GF (2019). Blood steroids are associated with prognosis and fat distribution in endometrial cancer. Gynecologic oncology.

[18] Yang R, Yang S, Li R (2016). Effects of hyperandrogenism on metabolic abnormalities in patients with polycystic ovary syndrome: a meta-analysis. Reproductive biology and endocrinology: RB&E.

[19] Piltonen TT (2016). Polycystic ovary syndrome: Endometrial markers. Best practice & research Clinical obstetrics & gynaecology.

[20] Kim SH, Kang HW, Jeong JB (2021). Association of obesity, visceral adiposity, and sarcopenia with an increased risk of metabolic syndrome: A retrospective study. PloS one.

[21] Qi J, Hu H, Yaghjyan L (2020). Association of Adipose Tissue Distribution with Type 2 Diabetes in Breast Cancer Patients. Breast cancer: basic and clinical research.

[22] Wang T, Zhang J, Hu M (2019). Differential Expression Patterns of Glycolytic Enzymes and Mitochondria-Dependent Apoptosis in PCOS Patients with Endometrial Hyperplasia, an Early Hallmark of Endometrial Cancer, In Vivo and the Impact of Metformin In Vitro. International journal of biological sciences.

[23] Ciortea R, Mihu D, Mihu CM (2014). Association between visceral fat, IL-8 and endometrial cancer. Anticancer research.

[24] Wiwatpanit T, Murphy AR, Lu Z (2020). Scaffold-Free Endometrial Organoids Respond to Excess Androgens Associated with Polycystic Ovarian Syndrome. The Journal of clinical endocrinology and metabolism.

[25] Ferreira SR, Motta AB (2018). Uterine Function: From Normal to Polycystic Ovarian Syndrome Alterations. Current medicinal chemistry.

[26] Freuer D, Linseisen J, O'Mara TA (2021). Body Fat Distribution and Risk of Breast, Endometrial, and Ovarian Cancer: A Two-Sample Mendelian Randomization Study. Cancers.

[27] Pasquali R (2018). Metabolic Syndrome in Polycystic Ovary Syndrome. Frontiers of hormone research.

[28] Polak K, Czyzyk A, Simoncini T (2017). New markers of insulin resistance in polycystic ovary syndrome. Journal of endocrinological investigation.

[29] Lim SS, Kakoly NS, Tan JWJ (2019). Metabolic syndrome in polycystic ovary syndrome: a systematic review, meta-analysis and meta-regression. Obesity reviews: an official journal of the International Association for the Study of Obesity.

